# Vision loss, vision difficulty and psychological distress in South Africa: results from SANHANES-1

**DOI:** 10.1186/s40359-021-00558-x

**Published:** 2021-04-29

**Authors:** Kwadwo Owusu Akuffo, Ronel Sewpaul, Samson Darrah, Natisha Dukhi, David Ben Kumah, Eldad Agyei-Manu, Emmanuel Kofi Addo, Akosua Kesewah Asare, Isaiah Osei Duah, Priscilla Reddy

**Affiliations:** 1 Department of Optometry and Visual Science, College of Science, Kwame Nkrumah University of Science and Technology, Kumasi, Ghana; 2 Health & Wellbeing, Human and Social Capabilities Division, Human Sciences Research Council, Cape Town, South Africa; 3 Usher Institute for Population Health Sciences and Informatics, College of Medicine and Veterinary Medicine, University of Edinburgh, Edinburgh, UK; 4 Department of Ophthalmology and Visual Sciences, Moran Eye Centre, University of Utah, Salt Lake City, Utah, USA; 5 Department of Nutrition and Integrative Physiology, University of Utah, Salt Lake City, Utah, USA; 6 Department of Ophthalmology and Visual Sciences, University of British Columbia, Vancouver, Canada; 7 Faculty of Health Sciences, Nelson Mandela University, Port Elizabeth, South Africa

**Keywords:** Vision-related quality of life, Emotional stress in eye diseases, Mental health, Anxiety, Depression, Vision impairment and stress, Vision disorders and self-esteem

## Abstract

**Background:**

Psychological distress in vision impairments and blindness is a complex issue and a major public health concern. Sudden adjustments in routine lifestyle and career aspirations in such persons culminate in and/or aggravate their level of stress. Yet, psychological distress in persons with visual difficulties and vision loss in South Africa is poorly understood. We investigated the association between psychological distress and self-reported vision difficulties as well as clinician-assessed vision loss using data from the South African National Health and Nutrition Examination Survey (SANHANES-1).

**Methods:**

Data was analysed on participants aged ≥ 15 years who participated in the SANHANES-1 clinical examinations and interviews. Data on demographic, socio-economic, and health status variables were gathered using a structured questionnaire. Psychological distress was assessed using the Kessler psychological distress scale (K10). Vision assessment was conducted by clinicians adhering to standard protocols as well as by participants’ subjective response to vision-related questions. Vision loss was defined as presenting visual acuity worse than Snellen 6/12 in the better eye. Bivariate and multiple logistic regressions were used to examine the association between vision parameters and psychological distress.

**Results:**

The analytic sample comprised 6859 participants with mean age of 38.4 years (60.8% females). The prevalence of psychological distress was 19.9%. After adjusting for demographics, socioeconomic, health risk and eye care variables, self-reported myopia (mild adjusted odds ratio [AOR] = 1.9, 95% CI 1.3–2.7; moderate AOR = 2.4, 95% CI 1.6–3.7; severe AOR = 3.6, 95% CI 1.8–7.3) and self-reported hyperopia (mild AOR = 1.7, 95% CI 1.2–2.5; moderate AOR = 2.4, 95% CI 1.5–3.8; severe AOR = 3.5, 95% CI 1.8–6.8) were significantly associated with psychological distress. While psychological distress was higher in patients with clinician assessed vision loss than those with normal vision, the association was not statistically significant after adjusting for confounders (AOR: 1.0, 95% CI 0.7–1.4).

**Conclusions:**

Persons who self-reported vision difficulty experienced a higher prevalence of psychological distress. Therefore, comprehensive psychological care is needed for patients with eye disease or vision difficulties as part of a governmental strategy to provide mental health care for all South Africans.

## Introduction

Vision loss arises as a result of pathologic or physiological changes in the cellular and molecular composition of the visual system either by aging, trauma, or diseases. Clinically, vision loss may manifest as difficulty and/or decreased visual performance on a standard visual acuity (VA) test chart [1]. According to the International Classification of Diseases (ICD), vision loss is broadly categorized into distance (poorer than Snellen 6/12 VA) and near (worse than N6 or M.08) visual impairment [2]. Humans interact with the world mainly through sight; with over eighty percent of surrounding information accessible to the brain circuitry via the eyes [3–5]. Undeniably, vision difficulty from impairment or blindness affects a person's physical and psychological well being, overall health, and quality of life [6–8].

Previous studies indicated that vision impairment and blindness influence the psychometric parameters in affected persons [9–12]. A systematic review, by Khoo [10] indicated that individuals with diabetes-related vision loss had poorer psychosocial outcomes as they suffer from anxiety, and depressive disorders, as well as problems with their emotional and social health. Berman et al. [9] also found a higher level of anxiety, depression, emotional distress, and increased mortality among the aged with functional visual impairment as a result of age-related macular degeneration.

Despite vision loss being an important determinant of psychological distress, a condition characterized by emotional suffering with accompanying symptoms of anxiety, depression and nervousness, its prevalence is alarmingly high among the South African populace [13]. Also, mental distress exacerbates the treatments and prognosis of ocular diseases and cause ascendency in the burden of vision loss [14]. Early diagnosis and treatment of vision loss has the potential to reduce the prevalence of mental health [15]. Contrastingly, identification of vision-related mental distress and provision of better coping strategies and assistance have been shown to improve visual outcomes and quality of life [16]. It is worth mentioning that the failure to recognize this as an important public health issue could result in secondary depression among victims and ultimate suicide. Nevertheless, there is a paucity of data to transform policy development and improve mental care among ophthalmic patients in South Africa.

This study investigated the association between self-reported vision difficulty, clinician-assessed vision loss, and psychological distress in persons aged 15 years and above, using data from the South African National Health and Nutrition Examination Survey [17]. Past previous studies exclusively focused on the association between vision loss and psychological distress in only older adults, particularly in high-income countries. Also, most of these studies were limited to the assessments of participants by clinicians and not by participants’ subjective response to vision-related questions. Given that one tenth of South Africans experience vision loss [13], the outcomes of this nationwide study may provide substantive evidence to accentuate the need for mental health assessment for the visually impaired/blind. Furthermore, this study will contribute substantially to raise awareness of the importance of addressing co-occurring visual disorders and psychological health problems in South Africa, which will improve the quality of life of affected individuals.

## Methods

### Study design, setting, and participants

**Fig. 1:**
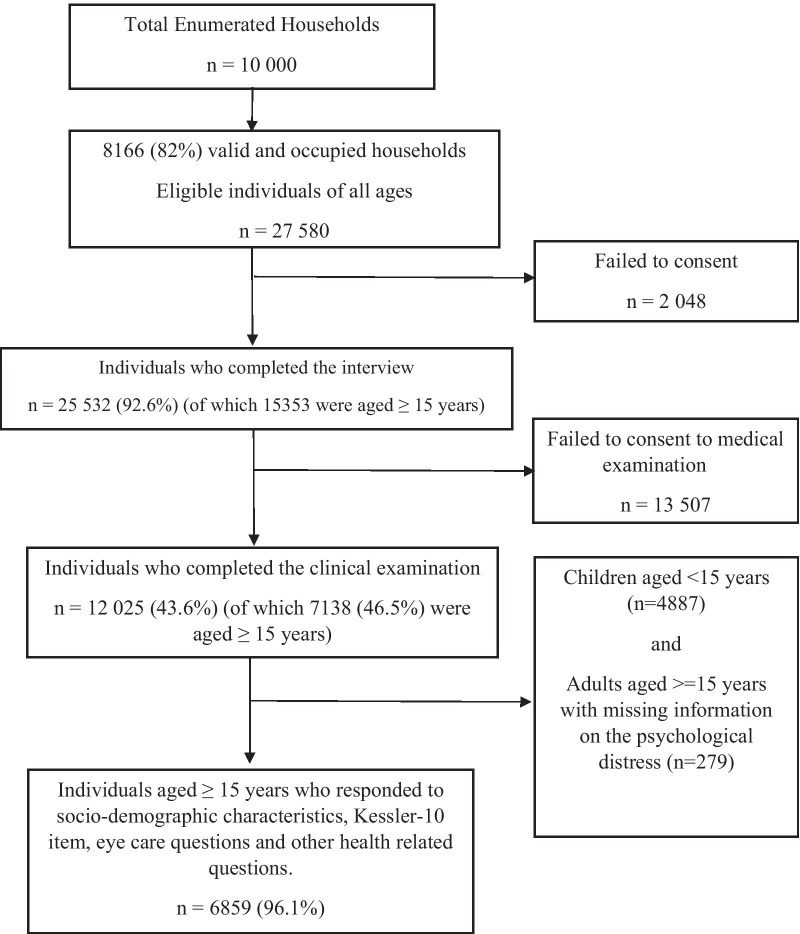
Flowchart showing participant enrollment, eligibility, assessment and the analytic sample

This study utilized data from SANHANES-1 [17], which enrolled participants of all ages. In brief, SANHANES-1 applied a stratified, multi-stage disproportionate cluster sample approach to select a total of 10,000 households within the Enumeration Areas (EAs) stratified by province and locality type. A total of 27,580 eligible individuals of all ages occupied 8166 valid households out of the 10,000 households, of which 25,532 (92.6%) participated in the interview. In addition, 12,025 (43.6%) individuals consented to undergo a clinical examination. Questions on vision difficulty and psychological distress were administered to participants aged ≥ 15 years. The final analytic sample comprised 6859 participants aged ≥ 15 years who underwent a physical examination (including vision assessments) and responded to the questions on vision difficulty, psychological distress, health status and sociodemographic characteristics. The demographic characteristics of the sample who volunteered to undergo a clinical examination differs slightly from the full interview sample. Therefore, the analytic sample comprises a higher proportion of females (60.8% vs. 54%) and rural participants (44% vs. 36%) and a lower proportion of participants aged 15–44 (66.3% vs. 71.1%) than the full sample. Details of SANHANES-1 eligibility, methodology, examination procedures and derivation of the analytic sample are summarized in Fig. 1.

### Ethical approval

The Research Ethics Committee of the South African Human Sciences Research Council (HSRC) approved the study (REC number: 6/16/11/11). All procedures employed in the study were in adherence with the tenets of the Declaration of Helsinki. Written informed consent/assent was obtained from all the survey participants. In addition, written informed consent/assent of parents/guardians was also obtained for children aged ≤ 17 years.

### Measures

Sociodemographic variables in our analysis included sex, age (15–44, 45–54, 55–64, or ≥ 65 years), and population group (African, White, Coloured, Indian/Asian). Of note, the term Coloured refers to individuals with mixed race (i.e., Mixed European and African or Asian ancestry) and is used in all national statistical reporting [18]. Socioeconomic characteristics included the highest education level (no formal schooling/grades 0–7, grade 8–12, and higher education), wealth index [1 (lowest wealth)-5 (highest wealth), and residence (rural/urban)].

#### Assessment of visual function

Well trained and equipped survey and clinical teams comprising of interviewers, a medical doctor, a registered nurse and a clinic assistant were involved in this assessment. Survey staffs conducted interviews and clinical examinations were performed by the clinical team. The primary independent variables were self-reported difficulty in seeing objects close-up (hyperopia) and self-reported difficulty seeing objects at a distance (myopia), and clinician assessed vision loss. Using the Snellen chart, the medical doctor assessed the subjects’ visual acuity to ascertain whether the participant had vision loss and if so, the type of vision loss. The type of vision loss was categorized into blurred vision, a need for more light, difficulty reading, loss of peripheral vision, difficulty driving at night, double vision, difficulty in distinguishing colours, straight lines looking wavy, and sensitivity to glare. The categories were not mutually exclusive, that is, a participant could experience multiple types of vision difficulties. Clinician assessed vision loss was defined as presenting visual acuity (PVA) worse than Snellen 6/12 in the better eye. For self-reported visual difficulties, self-reported myopia was assessed by the question: “In the last 30 days, how much difficulty did you have in seeing and recognizing an object or a person you know across the road (from a distance of about 20 m)”, where the participant was asked to answer including times when wearing glasses/contact lenses if used; with options for none, mild, moderate, and severe and extreme/cannot do. Similarly, self-reported hyperopia was measured by the question: “In the last 30 days, how much difficulty did you have in seeing and recognizing an object at arm’s length (for example, reading) where the participant was asked to include when wearing glasses/contact lenses if used.” In addition, self-reported use of eyeglasses or contact lenses to see things close up or far away such as when reading newsprint or identifying someone far away was obtained. The interview questions on self-reported vision difficulties were from the World Health Organization’s (WHO) Study on global ageing and adult health (SAGE), which was conducted in several countries, including in South Africa in 2007 [19]. Participants were also asked when last they had their eyes examined. All doctors recruited in the study were trained in standardized procedures of measuring visual acuity.

#### Psychological measures

The primary dependent variable was psychological distress, which was measured using the Kessler-10 psychological distress scale (K-10) [20]. The scale consists of 10 items (e.g., ‘In the past 4 weeks, about how often did you feel nervous that nothing could calm you down?’) where each item has five-level response scale: ‘all of the time’ (5), ‘most of the time’ (4), ‘some of the time’ (3), ‘a little of the time’ (2), and ‘none of the time’ [17]. The total score of the scale ranges between 10 and 50 where a score < 20 indicates low/minimal distress, a score from 20–24 indicates mild distress, a score from 25 to 29 indicates moderate distress, and a score ≥ 30 signifies severe distress [20]. Prior research indicates that the Kessler-10 scale relates with the Composite International Diagnostic Interview (CIDI) questionnaire which is now the standard tool for the assessment of mental disorders. This makes Kessler 10 scale a good tool for the assessment of psychological distress [21]. The scale has been validated in the South African context [22].

#### Hazardous alcohol use

Hazardous drinking was assessed using a three-item alcohol screening tool, the Alcohol Use Disorders Identification Test-Consumption (AUDIT-C) [23].

#### Tobacco use

Tobacco smoking status of participants (current smoker, ex-smoker, never smoker) was measured by self-reported current and past tobacco smoking.

#### Experience of traumatic event

For the assessment of the experience of any traumatic event, participants responded either yes or no to fourteen (14) listed events with a preamble ‘have you ever experienced any of the following events’ (for instance, ‘severe automobile accidents’ and ‘learned about the sudden, unexpected death of a family member or a close friend?’) [17].

#### Assessment of physical ill-health conditions

History of cardiac disease was assessed by participants’ self-report of whether a medical officer or health worker had ever told them that they have any of these conditions: heart (cardiac) disease, heart failure, stroke, rheumatic heart disease, a heart attack or chest pain (angina) [17] Diabetes was assessed by self-report of previous diagnosis of high blood sugar or diabetes by a health professional. Blood pressure was measured during the clinical examination. Hypertension was defined as having systolic blood pressure ≥ 140 mmHg, diastolic blood pressure ≥ 90 mmHg or current use of hypertensive medication.

### Data analysis

We analyzed data using Stata 15.0. (StataCorp, Texas, USA, 2016). The analyses utilized sample weights to adjust for unequal probabilities of selection and nonresponse as well as for the complex survey design using the ‘svy’ commands in Stata. Descriptive statistics were used to summarize the demographic, socioeconomic, health status and eye care characteristics. Chi-square tests were used to test the difference between estimates of psychological distress and level of vision difficulty and vision loss. A series of multiple logistic regression models were used to investigate the association of self-reported vision difficulties (both myopia and hyperopia) and clinician assessed vision loss with the binary outcome; mild to severe psychological distress. The binary outcome was coded by dichotomizing the Kessler-10 scale into two categories with a total score < 20 for no or minimal psychological distress (coded 0) and ≥ 20 for mild to severe psychological distress (coded 1) [24]. The following variables were added to each model: Model (1) adjusted for age, sex and population group; Model (2) Model 1 plus the socioeconomic variables (education, wealth quintile and urban/rural residence), Model (3) Model 2 plus the health status variables (tobacco smoking status, hazardous alcohol drinking, BMI, diabetes, hypertension, cardiac disease and lifetime experience of traumatic event(s) and Model (4) Model 3 plus use of a visual aid and years since last eye examination. The selected variables were included in the models as possible confounders based on a review of the literature, as they have been shown to have associations with both psychological distress and vision loss. Odds ratios (OR) with 95% confidence intervals were calculated. All estimates were considered statistically significant at *p* < 0.05.

## Results

### Participants and descriptive data

Table 1 presents the sociodemographic characteristics of the study participants. The analytic sample comprised 6859 participants (Fig. 1) with a mean (SE, standard error) age of 38.4 (0.35) years, with females accounting for 60.8% of the sample. The largest population group was African (83.6%), followed by Coloured (mixed-race) (10.7%), White (4.3%), and Indian (1.5%). Most of the participants (66.3%) were between 15 and 44 years old, majority (62.5%) had secondary school education (grade 8–12 or equivalent) and 55.5% lived in urban areas. The majority of the participants (79.6%) never smoked, and 18.6% were hazardous drinkers. Many had non-communicable diseases such as hypertension (32.7%), cardiac disease (10.3%), diabetes (6.5%), and 22.5% had experienced at least one traumatic event.

**Table 1:** Description of the sample

	%	95% CI	Frequency
Total	100		6859
*Demographic characteristics*			
Sex			
Males	39.2	37.6–40.8	2415
Females	60.8	59.2–62.4	4444
Age (Mean, SE)	38.4	0.35	
15–44 years	66.3	64.5–68.0	4062
45–54 years	13.8	12.6–15.0	1082
55–64 years	10.3	9.3–11.4	908
≥ 65 years	9.7	8.5–11.1	807
Population group			
African	83.6	80.5–86.3	4886
White	4.3	2.8–6.3	142
Coloured (Mixed-race)	10.7	8.6–13.2	1471
Indian	1.5	1.0–2.1	329
*Socioeconomic characteristics*			
Highest Education			
No formal schooling/Gr 0–7	30.4	28.0–32.9	2189
Grade 8–12 (or equivalent)	62.5	60.1–64.8	3813
Higher education	7.1	5.7–8.9	338
Wealth index			
Lowest (1)	24.2	20.6–28.2	1347
2	23.2	20.7–25.9	1282
3	22.5	19.9–25.4	1274
4	16.5	14.1–19.1	1029
Highest (5)	13.5	10.7–16.9	688
Residence			
Rural	44.5	38.6–50.6	2977
Urban	55.5	49.4–61.4	4465
*Health status variables*			
Tobacco smoking			
Never smoker	79.6	77.8–81.2	5208
Ex-smoker	4.3	3.6–5.1	268
Current smoker	16.2	14.6–17.8	1280
Hazardous alcohol use	18.6	16.8–20.5	1215
Diabetes	6.5	5.8–7.3	519
Hypertension	32.7	30.9–34.5	2552
Cardiac disease	10.3	9.2–11.5	688
Lifetime experience of ≥ 1 traumatic event	22.5	19.9–25.3	1327
*Eye care variables*			
Uses eye glasses or contact lenses	17.9	15.9–20.1	1217
Years since eyes examined			
< 1 year	6.1	4.9–7.6	354
1–2 years	8.8	7.5–10.2	592
3–5 years	4.4	3.7–5.2	323
> 5 years	3.3	2.7–4.0	220
Never	62.9	59.8–65.8	4134
Don't know	14.5	12.4–17.0	1025
Self-reported distance visual impairment			
None	80.3	78.6–81.9	5209
Mild	9.1	8.2–10.2	644
Moderate	7	6.2–7.9	482
Severe/Extreme/unable	3.5	2.8–4.4	252
Self-reported near visual impairment			
None	80.2	78.7–81.8	5206
Mild	10	9.0–11.1	708
Moderate	6.3	5.5–7.3	428
Severe/Extreme/unable	3.4	2.8–4.2	245
Clinician assessed vision loss	10.7	9.2–12.3	835

%, percentage of frequency; CI, confidence interval

The percentage of participants who used either eyeglasses or contact lenses was 17.9%, and a large proportion of the participants (62.9%) had never had their eyes examined. The majority of participants (80.3%) reported experiencing no difficulty with seeing at far distances (i.e., hyperopia), 9.1% had mild difficulty, 7% had moderate difficulty, and 3.5% had severe difficulty. Furthermore, 80.2% reported no difficulty with seeing at near distances (i.e., myopia), 10% had mild difficulty, 6.3% had moderate difficulty, and 3.4% had severe difficulty with hyperopia. Also, upon clinical assessment, 10.7% of the participants had distance vision loss.

Table 2 shows the prevalence of psychological distress by self-reported and clinician assessed vision difficulty. Overall, the prevalence of psychological distress in our study was 19.9% (mild, 11.6%; moderate, 5%; severe, 3.3%). For self-reported myopia, those who reported severe (extreme) vision difficulty experienced a very high psychological distress (K-10 score ≥ 30) (16.1% (95% CI 10.8–23.4%) whereas those who reported not having any visual difficulty experienced a very low psychological distress (K-10 score ≤ 19), (84.4% (95% CI 82.5–86.1%). Likewise, for self-reported hyperopia, participants who reported severe (extreme) vision difficulty experienced a very high psychological distress (K-10 score ≥ 30), 13.0% (95% CI 7.9–20.6%) while those who reported not having any vision difficulty experienced a very low psychological distress (K-10 score ≤ 19), 84.2% (95% CI 82.2–85.9%). Also, the prevalence of severe psychological distress was 4.5% (95% CI 3.1–6.5%) among participants who had vision loss upon assessment by clinicians, compared to 3.0% (95% CI 2.4–3.9%) among those who had normal vision. There were significant bivariate associations with the level of psychological distress and each of self-reported myopia, self-reported hyperopia and clinician assessed vision loss (*p* < 0.001).

**Table 2:** Prevalence of psychological distress by self-reported vision difficulty and clinician assessed vision loss

Level of psychological distress									
	Low distress (K10 ≤ 19)		Mild distress (K10 20–24)		Moderate distress (K10 25–29)		Very high/Severe distress (K10 ≥ 30)		*p* value (Chi-sq test)
	%	95% CI	%	95% CI	%	95% CI	%	95% CI	
*Total*	80.2	78.3–82.0	11.6	10.3–12.9	5	4.2–5.9	3.3	2.6–4.0	
*Self-reported myopia*									
None	84.4	82.5–86.1	9.7	8.5–11.1	3.5	2.7–4.4	2.4	1.8–3.1	< 0.001
Mild	70	64.6–74.9	17.3	13.6–21.7	8.9	6.2–12.7	3.8	2.4–6.0	
Moderate	59.5	52.9–65.8	21.2	16.6–26.6	12.9	9.2–17.7	6.5	4.2–9.8	
Severe/Extreme/unable	54.2	44.6–63.4	17.6	11.5–26.0	12.1	7.7–18.5	16.1	10.8–23.4	
*Self-reported hyperopia*									
None	84.2	82.2–85.9	9.8	8.6–11.2	3.7	3.0–4.6	2.3	1.8–3.0	< 0.001
Mild	71	66.0–75.6	17.8	14.4–21.7	7	4.8–10.0	4.2	2.5–7.1	
Moderate	59.6	53.1–65.8	19.8	15.6–24.7	12.1	8.4–17.2	8.5	6.0–11.8	
Severe/Extreme/unable	53	44.2–61.6	19.2	14.0–25.6	14.9	9.9–21.9	13	7.9–20.6	
*Clinician assessed vision loss*									
Normal	81.1	79.1–83.0	11.3	10.1–12.6	4.6	3.8–5.5	3	2.4–3.9	< 0.001
Loss of vision	71.9	67.3–76.1	15.9	12.2–20.4	7.7	5.6–10.5	4.5	3.1–6.5	

Table 3 shows multiple regression results for the association between self-reported myopia, self-reported hyperopia, and clinician assessed vision loss with psychological distress. For self-reported myopia, after adjusting for all sociodemographic, socioeconomic, health status and eye care variables (model 1–4), it was observed that participants who reported having mild (Adjusted odds ratio (AOR): 1.9, 95% CI 1.3–2.70, moderate (AOR: 2.4, 95% CI 1.6–3.7) or severe (AOR: 3.6, 95% CI 1.8–7.3) myopia were significantly more likely to experience psychological distress than those who reported having no myopia. Similarly, participants who reported mild (AOR: 1.7, 95% CI 1.2–2.5), moderate (AOR: 2.4, 95% CI 1.5–3.8) or severe (AOR: 3.5, 95% CI 1.8–6.8) hyperopia were more likely to experience psychological distress than those who reported no hyperopia. However, the association between psychological distress and loss of vision, as assessed by a clinician, was reduced and was no longer statistically significant after adjustment for age, sex and population group in Model 1. In Model 4, there remained no statistically significant association between having loss of vision, as assessed by a clinician, and psychological distress (AOR: 1.0, 95% CI 0.7–1.4).

**Table 3:** Multiple regression showing association of self-reported vision difficulty and vision loss with psychological distress^a^

	Model 1^b^		Model 2^c^		Model 3^d^		Model 4^e^	
	OR	95% CI	OR	95% CI	OR	95% CI	OR	95% CI
*Self-reported myopia*								
None	Ref	–	Ref	–	Ref	–	Ref	–
Mild	1.9	1.3–2.7	1.9	1.3–2.7	1.7	1.2–2.5	1.9	1.3–2.7
Moderate	2.6	1.7–3.9	2.7	1.8–4.0	2.3	1.5–3.4	2.4	1.6–3.7
Severe/extreme/unable	4.2	2.2–7.7	3.9	2.1–7.2	3.2	1.6–6.3	3.6	1.8–7.3
*Self-reported hyperopia*								
None	Ref	–	Ref	–	Ref	–	Ref	–
Mild	1.8	1.3–2.7	1.8	1.3–2.5	1.6	1.1–2.3	1.7	1.2–2.5
Moderate	2.4	1.6–3.7	2.5	1.6–4.0	2.2	1.3–3.5	2.4	1.5–3.8
Severe/extreme/unable	3.7	2.2–6.4	3.8	2.1–6.8	3.3	1.7–6.2	3.5	1.8–6.8
*Clinician assessed vision loss*								
Normal	Ref	–	–	–	–	–	–	–
Loss of vision	1.1	0.8–1.5	1.1	0.8–1.5	1	0.8–1.4	1	0.7–1.4

^a^Defined as a score of 20 or more on the Kessler Psychological Distress scale. ^b^Adjusted for age, sex, and population group. ^c^Model 1 plus education, wealth quintile and urban/rural residence. ^d^Model 2 plus tobacco smoking status, hazardous alcohol drinking, BMI, diabetes, hypertension, cardiac disease and lifetime experience of traumatic event(s). ^e^Model 3 plus use of a visual aid and years since last eye examination

*OR* odds ratio, *CI* confidence interval

## Discussion

This study investigated the association between clinician-assessed vision loss, self-reported vision difficulty, and psychological distress using a nationally representative sample from South Africa [17]. After adjusting for potential confounders, we found a statistically significant association between self-reported vision difficulty and psychological distress, where the higher the level of self-reported vision difficulty the higher the likelihood of experiencing psychological distress.

Having good vision is crucial in life and ultimately for survival. In performing day-to-day activities (such as reading, writing, threading needle, driving, etc.) an efficient vision is critical towards proficient productivity. Consequently, low vision and/or visual impairment defined as vision loss that cannot be corrected with medical/surgical intervention or optical corrections and that poses significant limitation in the normal discharge of activities of daily living, culminates in physical dependency, decreased performance, and ultimately poor financial status. Additionally, most professions require a higher level of visual acuity for enrolment, hence, any pathological changes that causes visual acuity to fall below the standard may result in denial and/or termination of job appointment. Given, the concurrent burden associated with vision loss, we provide additional evidence from a bio-behavioral nationwide survey to corroborate the hypothesis that vision difficulties relate to psychological distress.

In our study, we found an association between psychological distress and self-reported vision difficulty which persisted after adjustment of demographic, socioeconomic, health risk and eye care variables. This finding is consistent with findings from several studies [9, 11, 25, 26]. For example, Abateneh et al. [11] investigated the association between vision loss and psychological distress among outpatients attending the eye clinic in Rural Southwest Ethiopia and found a higher prevalence of psychological distress in persons with vision loss compared to those with normal vision. In a hospital-based cross-sectional study in the Netherlands, Van der Aa et al. [25] found a significantly greater degree of anxiety and depressive disorders among visually impaired persons compared to normal sighted persons. Similarly, a longitudinal study using data from Korean Health Insurance Review and Assessment Services, Lee et al. [26] found an increased risk of depression in both the nonblinded and blinded visually impaired groups, however, it was more pronounced in the latter groups than the former. The increased level of psychological distress found among persons with self-reported vision difficulties in our study may be attributable to their decreased comfortability and or deprivation in performing activities of daily living; notably, driving a car, reading a label on a medicine, writing a cheque, climbing stairs and recognizing faces [27].

Nonetheless, other population-based and longitudinal studies also reported no association between vision loss and psychological distress [28–30] which supports our study findings. For instance, a cross-sectional analysis of the twenty-five year follow up of the Wisconsin Epidemiologic Study of Diabetic Retinopathy, Hirai et al. [28] reported no association between the severity of diabetic retinopathy, visual impairment, and depression. A study by Forsell [30] that utilized data from the Swedish longitudinal study showed no association between vision impairment and the psychometric characteristics such as anxiety, depression, psychotic symptoms. Of note, these studies employed different psychometric metrics scale [Center for Epidemiologic Studies Depression Scale (CES-D), SHORT-CARE, Comprehensive Psychopathological Rating Scale (CPRS)] other than the Kessler 10 psychological distress scale used in our study. Self -reported difficulties in seeing objects at near or far distance reflect an individual’s perceived visual function and its impact on their daily activities, and the heightened stress or concern linked to this perception. Their perceptions summarize their overall visual ability, which comprises not only the loss of vision, but also other effects due to their perceived impairments. Some individuals clinically assessed as having vision loss may have found ways to cope with this impairment and may therefore exhibit depressive symptoms to a lesser degree.

Furthermore, previous studies focused exclusively on objective assessments of vision loss without considering participants’ subjective report [11, 31]. However, participants’ self-reported functional vision underscores the frequently assessed clinical measures including visual acuity. Thus, a person may have a reasonable visual acuity or function, but it may not be optimal in performing activities of daily living, and consequently culminates in psychological distress and associated symptoms [32]. Contrarily, fluctuations in psychological measures such as depression and anxiety in the visually impaired may affect objective clinical evaluation. Hence, listening and addressing the patient subjective concerns are of utmost importance given that their development of symptoms of emotional distress including depression is as a result of their unmet needs of providing treatment to their functional vision loss [33].

The study has several strengths. Several covariates were included to account for any potential biases by confounders and further evaluated demographic characteristics, socioeconomic characteristics, and health status variables consistent with previous studies. Additionally, we utilized a nationally representative sample, therefore the prevalence of psychological distress and vision difficulties can be generalized for the South African population. Furthermore, results from our study extend the existing literature by reporting the association between self-reported vision difficulty and psychological distress. This will inform policy formulation by relevant stakeholders and together help alleviate the burden of vision-related psychological distress in South Africa. Clinician assessed near visual acuity was not assessed in this study.

Nevertheless, the study has some limitations. The Kessler 10 psychological distress scale used for the assessment of psychological distress is a screening tool rather than a diagnostic one. Hence, the root cause of psychological distress is beyond the scope of this study. The cross-sectional nature of the study limits causational interpretations, and therefore longitudinal research is needed to improve the understanding of the relationship between visual difficulty and psychological distress.

## Conclusions

The high level of psychological distress among persons reporting vision difficulty implies that eye care professionals should know of the heightened risk of psychological distress among persons self-reporting vision problems. This finding calls for comprehensive psychological care, that includes screening, referral and treatment for depressive symptoms among patients with eye disease or vision difficulties, as part of a governmental strategy to provide mental health care for the populace. Additionally, mental health facilities should be made available and accessible to patients reporting vision difficulty.

Similarly, there is a need for large scale screening of South Africans to enable early detection and management of visual impairment. Studies have shown that older adults with refractive conditions had reduced depressive symptoms and better quality of life after receiving treatment or surgery for their conditions [34–36].

## Data Availability

The dataset(s) supporting the conclusions of this article is (are) available on request from the HSRC. The SANHANES data are available through registered access from the Human Sciences Research Council’s (HSRC) data repository at http://curation.hsrc.ac.za/Datasets-XKAHAA.phtml.

## Abbreviations

AOR: Adjusted odds ratio
AUDIT-C: Alcohol use disorders identification test-consumption
CIDI: Composite international diagnostic interview
HSRC: Human sciences research council
ICD: International Classification of Diseases
K-10: Kessler-10 psychological distress scale
PVA: Presenting visual acuity
REC: Research Ethics Committee
SANHANES: South African National Health and Nutrition Examination Survey
